# Human-induced land use changes and phosphorus limitation affect soil microbial biomass and ecosystem stoichiometry

**DOI:** 10.1371/journal.pone.0290687

**Published:** 2023-08-30

**Authors:** Johnny Kofi Awoonor, Bright Fafali Dogbey, Ibrahim Salis

**Affiliations:** 1 Soil Genesis, Survey and Classification Division, CSIR-Soil Research Institute, Kumasi, Ghana; 2 Department of Soil Resources Management, CSIR-College of Science and Technology, Kumasi, Ghana; 3 Department of Chemistry, School of Engineering, University for Development Studies, Tamale, Ghana; Government College University Faisalabad, PAKISTAN

## Abstract

Soil and microbial biomass carbon (C), nitrogen (N), and phosphorus (P) play an important role in soil nutrient dynamics in biogeochemical cycles of terrestrial ecosystems. However, increased human activities as a result of agricultural intensification on soil nutrients and microbial C:N:P stoichiometry are poorly understood in this fragile forest-savanna transition agroecosystem. This study aimed to (i) assess soil and microbial C, N, and P stoichiometry in different land use systems, and (ii) examine the effect of soil and microbial C, N, and P stoichiometry on soils susceptible to human-induced land use changes. A total of 82 composite soil samples at a depth of 0–20 cm were sampled from forest, savanna, grassland, fallow and cropland for laboratory analysis. The results revealed that the concentrations of C, N, and P were low in Fallow and Cropland compared to other land use systems. Analysis of variance in microbial C, N, and P stoichiometric ratios revealed a significant decreasing tendency compared to soil C:N, C:P and N:P ratios with no statistical significance (p < 0.05). The C:P and N:P ratios were low compared to the C:N ratio in land uses. A significant positive correlation was observed between MBC and MBN (0.95; p < 0.01), and with C and N (0.69; p < 0.01). There were significant interactive effects of land use on soil and microbial variables. The estimated microbial C:N:P stoichiometric ratios (21:2:1) were well constrained in the study area. The transition from Forest to Cropland resulted in 64%, 52%, and 71% reduction in C, N, and P, respectively. This implies that phosphorus is the main factor limiting productivity. The low availability of phosphorus in these tropical soils may have resulted in low C:P and N:P ratios. Therefore, we conclude that our results highlight the importance of phosphorus limitation on ratios of microbial C:P and N:P in landuse systems. Nutrient inputs such as fertilizers, manure and crop residues should be applied to croplands to improve soil and microbial C, N and P levels. Further, effects of land use on soil nutrient status and stoichiometry at 1-meter depth will be considered in our future work.

## Introduction

In countries south of the Sahara desert, more than half of their soil resources are highly weathered, leached, and impoverished, and these soils require nutrient conservation mechanisms. Soil microbial biomass (SMB) is a fundamental indicator for evaluating soil fertility [1], and it drives the carbon (C), nitrogen (N), and phosphorus (P) nutrient cycles of agroecosystems [2, 3]. Research findings indicate that SMB plays a crucial role in soil nutrient transformation by acting as a labile nutrient pool available to plants [4–6]. This is affected by biotic and abiotic factors. The biotic (flora and fauna), constitute the living components present in an ecosystem. However, the abiotic factors (climate, humidity, rainfall, soil type, light penetration, altitude etc.) refer to the non-living (physical and chemical) components of an ecosystem. Based on our current understanding, SMB assessment is a valuable tool for forecasting the long-term effects of land-use change and for understanding microbial nutrient limitations in soils [7–9]. Furthermore, Li et al. [10], in a recent study observed that soil microbes play a significant role in global soil nitrogen mineralisation. Thus, nitrogen mineralisation influences microbial biomass and its elemental stoichiometric ratios [11].

Additionally, previous studies found that soil physicochemical properties, land use, field management practices, pollutants and pesticides affect the amount and functionality of microbial biomass [12–16]. Due to the interactions between long-term (climate and soil properties) and short-term (fertilisation and tillage) factors, it is challenging to quantify the influence of environmental factors on soil and microbial biomass carbon, nitrogen, and phosphorus. Thus, Ho et al. [17] and Lu et al. [18] stressed that microbes influence ecosystem carbon turnover, nutrient mineralisation, and ecosystem sustainability. Biological relationships and interactions are essential in soil microbial functions, making microbes collaborate to maximise their ecological roles [19–21]. Considered as the living component of organic matter, microbial biomass, according to Sousa et al. [22], is made up of microorganisms. Also, Sparling [23] observed that MBC constitute about 1–5% of organic matter compared to microbial nitrogen and phosphorus which constitute 2–10% of phosphorus [24].

Several research findings indicated that, ecosystem stoichiometry varies significantly among land use types [6, 25–27]. As a result, evaluating C:N:P stoichiometric patterns in land use types can improve our understanding of nutrient limitations in ecosystems under changing environments. Cleveland and Liptzin [28] indicated that C, N, and P ratios are linked to living organisms in the soil and can be used to indicate terrestrial nutrient limitation as well as assess ecological processes linked to litter decomposition [29]. Our current understanding indicates that the potential mechanism responsible for the variations in C:N:P stoichiometry in ecosystems and/or land uses is unclear. Soil micro-organisms possess unique elemental compositions and a change in microbial communities may result in elemental ratio differences in biomass [30]. Therefore, a change in phosphorus deficient tropical soils reveal a shift of the composition of microbial community in the plant litter microbe system [31, 32].

In Ghana, the dry and wet seasons are too extreme, affecting productivity, nutrient cycling, and microbial biomass quantity and quality. A review of available literature highlight the seasonal fluctuation of microbial biomass, with larger SMB contents in the dry season [33, 34] compared to the wet season [35]. In the dry season, plants delay growth and nutrient uptake resulting in the storage of microbial biomass as nutrients [36]. In the wet seasons, plant growth, root activity, and soil moisture increase, stimulating nutrient turnover by soil microbes in tropical biomes [37, 38]. According to Singh et al. [36], this results in a decrease in microbial biomass and an increase in CO_2_ efflux rates in tropical environments. In the major and minor dry seasons (high biomass, low turnover), when plant activity is intense, nutrients cannot be effectively extracted from the soil, and the most important role of microbial biomass is to store and conserve soil nutrients in a biologically active state. The release of these nutrient stocks during the major and minor rainy seasons (low biomass, high turnover) increases plant growth and development [39].

According to Bouma [40] and Lagerlof et al. [41], a change in land use affects soil function and significantly influences microbial biomass diversity and structure. An increase in anthropogenic perturbation has resulted in a decrease in carbon and nitrogen stocks by 25–30% in natural ecosystems. This has changed carbon and nitrogen turnover and sequestration in tropical agro-ecosystems [3, 42, 43]. Agricultural practices have altered soil microbial population, diversity and structure [44, 45]). Soil microorganisms play a critical role in mineralisation, immobilisation, nutrient cycling, and xenobiotic decomposition, resulting in a positive effect on soil physicochemical properties [46]. Xiao et al. [47] and Xue et al. [45] stressed the importance of soil pH as an essential indicator of soil microbial functions [45, 47]. In the Nkoranza district, the soil landscape is a fundamental land unit managed by smallholder farmers for agricultural production. Unfortunately, the study area is characterized by inappropriate agricultural practices and seasonal bush fires have resulted in severe soil erosion. Due to long periods of intensive cultivation, soil carbon, nitrogen and phosphorus exhibit a strong spatial variability at the landscape level [48].

In recent years, an increase in microbial biomass carbon, nitrogen and phosphorus has been emphasised as viable indicators of land-use change. Sparling [49] stressed that soil microbial biomass has a rapid turnover rate compared to soil organic matter. A valid approach to the evaluation of SMB is to compare data obtained from a native forest with cropland or fields under similar experimental conditions [50]. In Cleveland and Liptzin’s [28] study, soil and microbial biomass ratios revealed a C:N:P ratio of 186:13:1 in global soils and 60:7:1 in grassland and forest soils. However, evidence of temporal variability in microbial biomass is due to an increase in disturbance levels due to human activities, natural disasters, and long-term ecological differences in geology, topography, and climate [37]. Thus, the conversion of Forest to Cropland alters environmental conditions across microbial habitats in soils, and this decreases microbial activity and diversity.

Understanding the variability of soil C:N:P ratio is essential for identifying microbial limitations in soils. However, the variation in stoichiometry in heterotrophic microbes in soils has received limited attention in the Nkoranza district. Additionally, SMB is a crucial biological process that influences the immobilisation and mineralisation of available nutrients. Therefore, microbial biomass can serve as a sink (immobilisation) or a source (mineralisation) of nutrients, and this depends on carbon, nitrogen, and phosphorus expressed as C:N:P ratio. Ecological stoichiometry focuses on energy balance in ecosystems and the proportional relationship between soil chemical elements and nutrient cycling processes in ecosystems [51]. Also, limited research has been carried out on soil and microbial biomass carbon, nitrogen, and phosphorus and their stoichiometric relationship at the landscape level [52] in the Nkoranza district. Thus, these studies investigate the concentration of nutrient elements in soil and mostly neglect their stoichiometric ratios. However, improper soil resource management has led to soil degradation, threatening food security and ecosystem sustainability [48]. This is a major challenge as the population increases in this agroecological zone [53].

Several research findings stressed that phosphorus is a limiting nutrient in tropical soils, and very few studies have considered the impact of land-use change on available phosphorus [2, 54–56]. Furthermore, the interactions between land use and environmental factors on C:N:P stoichiometry of microbial biomass after land use change remain unknown in the Nkoranza district. According to Bationo and Fening [3], the rapid turnover rates of organic materials is due to high soil temperatures and fauna activities of termites. Recognising the potential of soils and their susceptibility to management under various local conditions requires our understanding of how microbial attributes can provide very useful information on land management and its variability across landscapes. Therefore, the objectives of this study were to: (i) assess the stoichiometry of C, N, and P in soils of different landuse systems, (ii) evaluate the effects of land use on microbial C, N, and P stoichiometric ratios across different land uses and (iii) examine the interactive effect of soil and microbial C, N, and P stoichiometry on soils susceptible to human-induced land use changes. We hypothesised that land uses with low carbon, nitrogen, and phosphorus stocks may have low C:N:P stochiometric ratios due to reduced carbon input. Also, it was hypothesized that land use change from forest to cropland influenced soil microbial stoichiometric and elemental ratios. Similarly, we hypothesised that the interactions of soil depth and land use on microbial C:N:P affected microbial biomass elemental stoichiometry between landuse types.

## Materials and methods

### Study area

**Fig 1:**
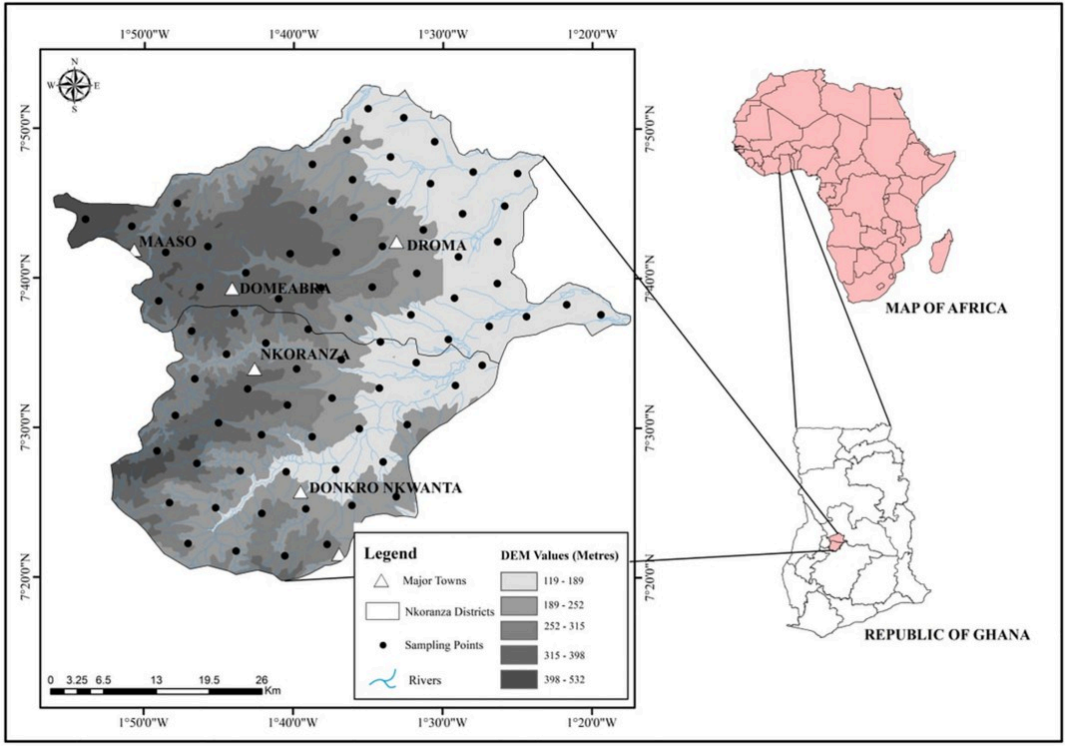
Illustrates location and elevation map of Nkoranza (north and south) with soil sampling points in the Bono East region of Ghana.

The study site is located in the Nkoranza (north and south) district, relatively situated in the centre of Ghana. The study area lies on longitude 1°10′ & 1°55′ West, and latitude 7°20′, 7°55′ North and covers approximately 2,592.09 km^2^ (Fig 1).

The site’s elevation ranged from 153 to 305 meters above sea level, with an annual temperature and precipitation of 36°C and 1600 mm, respectively. According to the Koppen-Geiger climate classification, the study area experiences the equatorial climatic regime with a tropical wet savannah climate (Aw) [57]. Potential evapotranspiration was 1400 mm per year. Relative humidity ranges from 90% to 95% and 75% to 80% in the rainy and dry seasons. The soils were classified as Lixisol (66%), Luvisol (9.54%), Gleysol (9.25%), Planosol (4.95%), Plinthosol (4.24%), while Acrisol, Fluvisol, Leptosol, and Nitisol contributed approximately 6%. Buri et al. [58] reported that most of the soils’ physical and chemical properties are generally low. The soils ranged from sandy loam to sandy clay loam in terms of texture. These soils were developed from sandstone and shale [2]. The soils were formed under the influence of ground, and lateral water flow from adjacent uplands. The area is characterised by extensive smallholder agricultural activity. The relatively high agro diversity reveals the transitional characteristics of prevailing ecosystems. This has resulted in the cultivation of crops adapted to humid and drier conditions. Land-use mainly includes forest, savannah woodland, grassland, fallow, and croplands (mostly maize fields) (Table 1).

**Table 1:** Dominant plant species in land use types in the study area.

Land use	Sample Size	Parent Material/ Geology	Dominant Soil Type	Description of land-use type	Age	Reference
Forest	11	Birimian sediment	Acrisol	This preserved area received less human interference. The land is covered with tall and dense trees. The forest was used as a reference. This system consists of native tree species including *Anogeissus leiocarpus, Borassus aetiopum, Parkia clappertoniana* and *Lophira alata*	50+	[59–61]
Savannah	14	Birimian sediments, Upper Voltaian, Obusum Oti bed	Acrisol, Luvisol, Lixisol, Gleysol, Plinthosol	This biome consists of a savannah vegetation mixed with woodland and grassland ecosystems. It is characterized by trees widely spaced. Sufficient sunlight reaches the ground given rise to micro-climate that support grasses. Savannah woodland is mainly made up of grasses *(Andropogon gayanus, Imperata cylindrical, Pennisetum purpureum) and trees (Anogeissus leiocarpus, Borassus aetiopum, Parkia clappertoniana, Lophira alata)*	30+	[59, 61]
Grassland	12	Obusum-Oti Beds, Upper Voltaian	Plinthosol, Gleysol, Lixisol, Planosol	A rolling terrain with grasses. The local climate favour grasses and in some cases a few trees. Grass species include *Andropogon gayanus, Imperata cylindrical, Pennisetum purpureum*	30+	[59, 61]
Fallow	22	Obusum-Oti Beds, Upper Voltaian	Acrisol, Gleysol, Plinthosol, Lixisol, Luvisol, Fluvisol	Most of these lands were abandoned for more done three years to recover its fertility. Dominant plant species include Chromolaena odorata (locally called Acheampong weed), spear grass *(Imperata Cylindrica L) Beauv, Cyperus rotundus, Eurhorbia heterophylla, Commenlina spp, Centrosema pubescens, Rottboellia cochinchinensis)*	<5	[61, 62]
Cropland	29	Dahomeyan, Obusum-Oti Beds, Upper Voltaian	Luvisol, Gleysol, Lixisol, Plinthosol	Land was cropped to maize continuously. This is a rain-fed cropping system. Continuous clearing, removal of above ground biomass (crop residue) and levelling of farm fields resulted in land-use change. The use of manure and fertilizer was low on these farms. Soil samples were sampled after harvest.	5+	[61]

### Soil sampling and description

**Fig 2:**
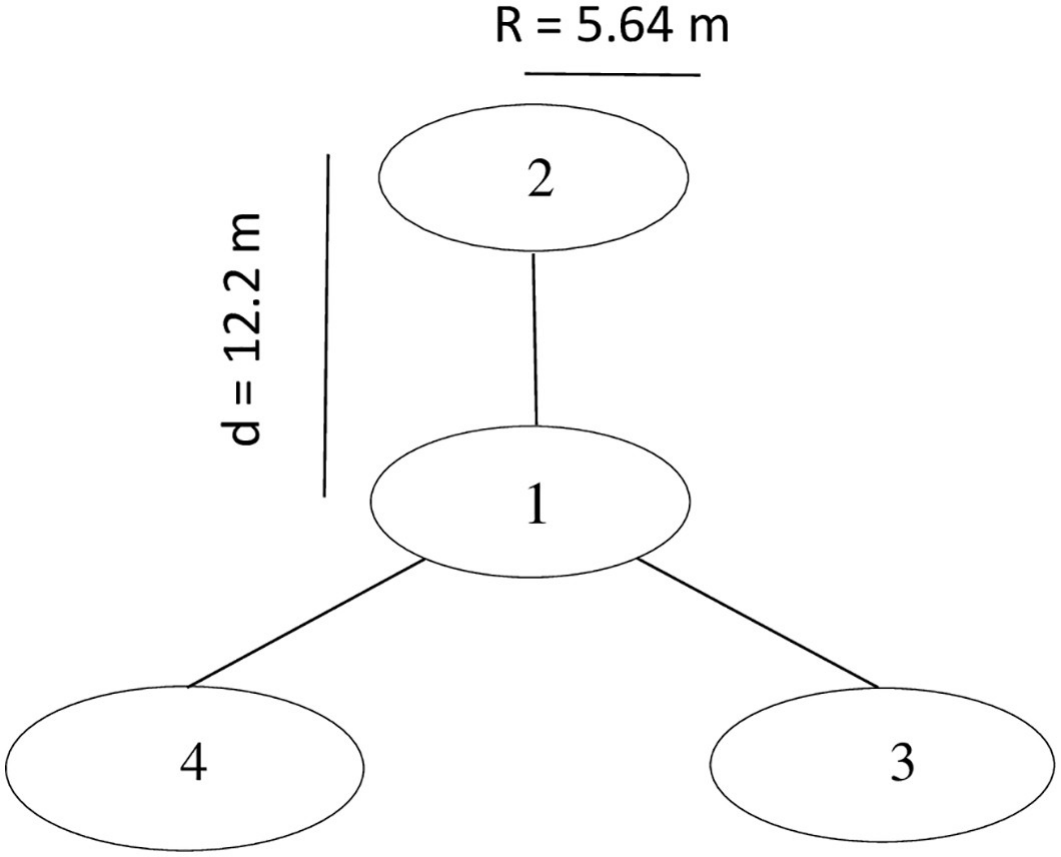
Illustrates soil sampling layout. Figs 1, 2, 3, and 4 indicates sampling subplots. At a distance of 12.2 metres from the centre point (sub-plot 1) up-slope, subplot 2 was marked. Subplots 3 and 4 were offset at 120 and 240 degrees down-slope, respectively. Also, R is the subplot radius and d is the distance between sub-plots centre-points [63].

A 25 km-grid soil sampling strategy was adopted using the fishnet tool in ArcGIS 10.6 (ESRI, Inc, USA). This sampling method was adopted due to the size of the study district (2,592.09 km^2^). The stratified sampling was used in the selection of 82 geo-referenced locations taken into consideration topography and the stratification of landuse. Eighty-two (82) composite soils were sampled from five (5) land-use types (Table 1). General information on land uses was obtained through interviews with local farmers. The selection of cropland sites depended on whether they had similar farming practices. Fertilizer use on croplands was low and differed from the farmlands sampled. Savannah woodland sites were selected from a large area with scattered trees and grasses. For Grassland, sites were selected from an area covered by extensive grasses and mostly grazed by livestock. All samples were collected from the 0–20 cm soil depth because it represents the average plough layer and most of the biological response of soil properties takes place in this layer compared to subsurface horizons. Landuse data for each sample point was superimposed on a digital elevation model (DEM) at a 50-metre spatial resolution. The Land Degradation Soil Sampling Framework (LDSF) developed by Vagen et al. [63] based on standard scientific principles was adopted (Fig 2). At 12.2 metres interval, soil samples were taken at each sample point using an auger (sampler size: 15 cm in length, 5 cm in diameter) at a depth of 20 cm.

This sampling method is suitable for smallholder farms (from 1–10 acres). Also, GPS coordinates were recorded using a hand-held Garmin Etrex 32x GPS (Garmin Ltd, USA). The interpretation of data requires the use of a reference standard. Soils from arable areas were compared to naturally-forested soils, where the natural ecosystem is fundamentally different. At each site, soil and microbial samples from sub-plots 1, 2, 3, and 4 (Fig 2) were composited, bagged, labelled and transported to the laboratory for analysis. Soil samples were air-dried and sieved through a 2 mm filter to determine the physical and chemical properties of the soil. To evaluate soil microbial biomass, soil water content of samples was adjusted to 40% field capacity, and pre-incubated for seven (7) days.

### Determination of soil chemical and biological properties

The modified version of the Walkley and Black titrimetric chromic acid wet oxidation method [64], as described by Nelson and Sommers [65], was used to estimate C. Total nitrogen (N) was determined by the Kjeldahl method [66] using a Kjeldahl distiller (Velp Scientifica UDK 139). Available phosphorus (P) was determined colourimetrically (UV-VIS Spectrophotometer + Peltier/Sipper System, PRIXMA UV-VIS 1800) after extraction with Bray’s No.1 solution [67]. Soil reaction (pH) was determined in distilled water at a soil:water ratio of 1:2.5 using a glass electrode and pH meter (BANTE 902) [68]. Soil microbial biomass C, N, and P concentrations were determined by chloroform fumigation extraction [69, 70]. Ten (10) grams of soil samples at field capacity, was put in a crucible and placed in a desiccator. A shallow dish containing 30 ml of alcohol-free chloroform was placed by it. Crucibles containing 10 g of soil samples were placed in a separate desiccator without chloroform to serve as control samples. The desiccators were covered and allowed to stand at room temperature for 5 days as described by Anderson and Ingram [71]. After the fumigation process, 50 ml of 0.5 M of K_2_SO_4_ solution was added to the soil samples to extract soil microbial carbon and nitrogen. Microbial biomass nitrogen was then determined by the Kjeldahl method. The amount of microbial carbon in the extract was determined using the colorimetric method. Five (5) ml of aliquot of the extract was pipetted into a 250 ml Erlenmeyer flask. Five (5) ml of 1.0 N (0.1667 M) potassium dichromate and ten (10) ml of concentrated sulphuric acid were added. The resulting solution was allowed to cool for 30 minutes after which 10 ml of distilled water was added. A standard series was developed concurrently with carbon concentrations of 0, 2.5, 5.0, 7.5, 10.0 mg ml^−1^ C. These concentrations were obtained while volumes of 0, 5, 10, 15 and 20 ml of a 50 mg ml^−1^ C stock were pipetted into 100 ml volumetric flasks and filled up to the mark with distilled water, respectively. The absorbance of the standard and sample solutions were read on a Spectronic 21D spectrophotometer at a wavelength of 600 nm. A standard curve was obtained by plotting the absorbance values of the standard solutions against their corresponding concentrations of the samples, as determined from the standard curve. Microbial biomass C was estimated as the difference between fumigated and the unfumigated samples using the conversion factors, 0.45 for C [72] and 0.45 for N [73] and 0.40 for P [72, 74]. Moreover, soil microbial stoichiometric ratios (microbial C:N, microbial C:P and microbial N:P) were then calculated on a molar basis.

### Statistical analysis

Data were checked for normality and homogeneity of variance to satisfy the assumptions of the statistical analyses. Where necessary, a log-transformation (base 10) was performed where the data did not show a normal distribution. Further, the effect of land use type on soil and microbial nutrient contents and stoichiometry were examined using a one-way analysis of variance (ANOVA). Multiple comparison tests were performed using Duncan’s multiple range test to identify significant effects on soil nutrient contents and stoichiometry between land use systems. The Pearson correlation coefficient was performed to quantify the relationships between land uses, soil, and microbial stoichiometry. Differences and correlations were considered statistically significant if p < 0.05 and highly significant if p < 0.01. We performed hierarchical cluster analysis using the square Euclidean distance method to identify similarity coefficients as the group connection algorithm to analyze soil and microbial characteristics. This assisted in the selection of the most appropriate divide based on the hierarchical tree [75]. All statistical analyses and drawing of figures were performed using GraphPad Prism 8 and OriginPro 2022 for windows, respectively.

## Ethics statement/approval

The CSIR-Soil Research Institute, Kumasi—Ghana, had a permit from Nkoranza District Assembly before soil sampling. This research was carried out in compliance with the laws of the Government of Ghana. The study did not involve the measurement of humans and animals. Also, no endangered or protected plant species was involved in the entire study.

## Results

### Soil C, N, and P stoichiometry

Table 2 shows the descriptive statistics of soil properties for each sampled site. The CV (%) results ranged from 0.36 under Cropland for soil pH to 121.99 mg kg^-1^ for P under Forest land use. These results showed a wide range of variations in soil properties within and across land-use types. Generally, soil pH ranged from 4.00 to 7.50, with a mean of 6.15. At a depth of 0–20 cm, mean soil pH values for Forest, Savannah, Grassland, Fallow and Cropland were 6.48, 6.35, 5.85, 6.20, and 5.88, respectively. Soil organic carbon ranged from 1.33 to 2.23% (Table 2). Mean values followed the order: Forest > Grassland > Savannah > Fallow > Cropland.

**Table 2:** Descriptive statistics of soil pH, C, N, and P concentration in land uses.

Variable	Land use	Min	Max	Mean	SD	CV (%)	Skewness	Kurtosis
pH (1:25)	Forest	6.10	7.00	6.48a	0.41	0.17	0.70	-1.65
	Savannah	5.20	7.50	6.35a	0.96	0.92	0.01	0.21
	Grassland	4.50	6.40	5.85a	0.90	0.82	-1.95	3.84
	Fallow	5.30	7.50	6.20a	1.05	1.10	0.59	-2.63
	Cropland	5.00	6.30	5.88a	0.60	0.36	-1.75	3.09
	All	4.50	7.50	6.15	0.77	0.60	-0.26	0.13
C (%)	Forest	2.37	3.83	3.01a	0.61	20.17	0.85	1.73
	Savannah	0.85	1.84	1.29c	0.41	31.93	0.72	1.26
	Grassland	1.97	2.48	2.22b	0.22	9.81	0.23	-0.64
	Fallow	0.83	1.54	1.17c	0.39	33.01	0.04	-5.78
	Cropland	0.61	1.45	1.06c	0.34	32.57	-0.47	1.62
	All	1.33	2.23	1.75	0.39	25.50	0.27	-0.36
N (%)	Forest	0.14	0.24	0.19a	0.04	21.71	-0.24	-0.79
	Savannah	0.06	0.13	0.11b	0.03	31.59	-1.10	-0.05
	Grassland	0.13	0.29	0.18b	0.08	42.50	1.93	3.77
	Fallow	0.05	0.17	0.10b	0.05	48.70	0.86	1.74
	Cropland	0.06	0.13	0.09b	0.03	35.72	0.23	-3.87
	All	0.09	0.19	0.13	0.05	36.04	0.34	0.16
P (mg kg^-1^)	Forest	3.51	62.19	22.69a	27.67	121.99	1.50	1.88
	Savannah	2.27	22.56	8.60a	9.40	109.26	1.89	3.64
	Grassland	1.04	40.82	15.29a	17.56	114.84	1.64	2.97
	Fallow	0.08	8.37	4.63a	3.55	76.81	-0.56	-0.31
	Cropland	2.07	9.65	6.42a	3.28	51.12	-0.83	0.13
	All	1.79	28.72	11.53	12.29	94.80	0.73	1.66

Abbreviation: Min., minimum; Max., maximum; SD, Standard deviation; CV, Cumulative variation. Different letters following mean values indicate a significant difference (p < 0.05) using a non-parametric multivariate comparison. Abbreviation: pH, soil pH; C, Soil organic carbon; N, Total nitrogen; P, Available phosphorus.

Available phosphorus (P) ranged from 1.79 mg kg^-1^ to 28.72 mg kg^-1^ at a mean of 11.53 mg kg^-1^, respectively. Within land use, P was in the order: Forest (22.69 mg kg^-1^), Grassland (15.29 mg kg^-1^), Savannah woodland (8.60 mg kg^-1^), Fallow (4.63 mg kg^-1^) and Cropland (6.42 mg kg^-1^). Also, C, N and P values in cropland were lower than the critical levels below which soil quality declined. Statistically, a significant difference was observed in C (p < 0.001), and N (p < 0.01) compared to soil pH and P in all land-use systems (Table 3). In terms of percentage change, soil C, N, and P decreased by 64%, 52%, and 71%, respectively due to land use change from forest to cropland. Land-use conversion produced a remarkable change in the stoichiometry of C, N, and P in the 0–20 cm soil depth. The C:N ratio ranged from 9.11 to 21.58, with a mean of 13.91 among land uses (Table 4). The highest C:N ratio was observed in Forest, and the lowest was measured in Fallow. Mean C:N ratio values of Cropland (12.77) and Fallow (12.67) land uses were close to each other compared to Forest, Savannah and Grassland with mean values of 15.81, 14.56, and 13.37, respectively. The highest and lowest C:P ratios were observed in Fallow (2.77) and Cropland (0.23), respectively. The N:P ratio decreased among land-use types: Fallow > Grassland > Forest = Savannah > Cropland. The estimated values for C:N, C:P and N:P ratios between nutrient concentrations were: Forest (15.81, 0.52, 0.03), Savannah (14.56, 0.26, 0.03), Grassland (13.73, 0.61, 0.04), Fallow (12.67, 2.77, 0.17), and Cropland (12.77, 0.23, 0.02). No significant difference was observed among land-use types for C:N, C:P and N:P ratio (p > 0.05). In terms of percentage from Forest to Cropland, ratios of C:N, C:P, and N:P decreased by 19%, 55%, and 33%, respectively. These ratios imply that the soils of the study area were well-constrained.

**Table 3:** Analysis of variance of soil C, N, and P stoichiometry.

Source	DF	pH	C	N	P	C:N Ratio	C:P Ratio	N:P Ratio
**1-Way ANOVA**								
Landuse	4	0.76	<0.001**	0.019*	0.472	0.874	0.522	0.567
Error		0.58	0.234	0.03	10.14	4.413	1.618	0.090

Values represent F values.

*p(F) < 0.01;

**p(F) < 0.001;

p(F) < 0.05).

Abbreviation: pH, soil pH; C, Soil organic carbon; N, Total nitrogen; P, Available phosphorus; C:N ratio, carbon and nitrogen ratio, C:P ratio, carbon and phosphorus ratio; N:P ratio, nitrogen and phosphorus ratio.

**Table 4:** Descriptive statistics of C, N, and P stoichiometry in land uses.

Variable	Land use	Min	Max	Mean	SD	CV (%)	Skewness	Kurtosis
C:N Ratio	Forest	12.42	18.24	15.81a	2.46	15.58	-1.07	1.71
	Savannah	8.50	30.67	14.56a	10.76	73.88	1.98	3.92
	Grassland	7.86	17.71	13.73a	4.19	30.48	-1.24	2.17
	Fallow	8.40	17.11	12.67a	4.83	38.13	0.01	-5.94
	Cropland	8.38	24.17	12.77a	7.62	59.68	1.96	3.87
	All	9.11	21.58	13.91	5.97	43.55	0.33	1.15
C:P Ratio	Forest	0.05	1.09	0.52a	0.53	100.20	0.17	-4.90
	Savannah	0.08	0.52	0.26a	0.18	70.15	1.15	2.08
	Grassland	0.06	1.89	0.61a	0.86	140.46	1.91	3.70
	Fallow	0.14	10.38	2.77a	5.07	182.93	2.00	3.99
	Cropland	0.10	0.53	0.23a	0.20	86.92	1.83	3.36
	All	0.09	2.88	0.88	0.37	116.13	1.41	1.65
N:P Ratio	Forest	0.01	0.07	0.03a	0.10	100.16	0.04	-5.64
	Savannah	0.01	0.06	0.03a	0.02	83.53	0.77	1.83
	Grassland	0.01	0.13	0.04a	0.06	138.24	1.96	3.88
	Fallow	0.02	0.63	0.17a	0.30	176.69	2.00	4.00
	Cropland	0.01	0.06	0.02a	0.03	112.73	1.97	3.89
	All	0.01	0.19	0.06	0.02	122.27	1.35	1.59

Different letters following mean values indicate a significant difference (P < 0.05) using a non-parametric multivariate comparison. Abbreviation: SD, Standard deviation; CV, Cumulative variance; C:N ratio, carbon and nitrogen ratio; C:P ratio, carbon and phosphorus ratio; N:P ratio, nitrogen and phosphorus ratio.

### Microbial C, N, and P stoichiometry

A cumulative variance of MBC, MBN, and MBP ranged from 5.00 to 48.58%. This reveals a wide variation in soil microbial properties (Table 5). A low CV(%) was obtained for MBC at all sites. However, the highest CV (%) was obtained from MBP under Grassland and MBN recorded the least under Cropland. Soil MBC ranged from 83.45 mg kg^-1^ to 128.15 mg kg^-1^. Within land-use systems, MBC followed the order: Forest > Savannah > Grassland > Cropland > Fallow (Table 5). Microbial biomass nitrogen ranged from 8.16 mg kg^-1^ to 10.97 mg kg^-1^. Among land-use types: Forest, Savannah, Grassland, Fallow, and Cropland recorded 17.66, 12.91, 7.23, 5.97, and 4.11 mg kg^-1^, respectively. While MBP ranged from 3.27 to 7.20 mg kg^-1^. Mean MBC, MBN, and MBP for the 0–20 cm were 106.79 mg kg^-1^, 9.58 mg kg^-1^ and 5.64 mg kg^-1^, respectively. However, MBP followed the order Forest > Grassland > Savannah > Fallow > Cropland thus 7.20, 6.89, 6.73, 4.13, and 3.27 mg kg^-1^, respectively (Table 5). Soil MBC (p < 0.001), MBN (p < 0.001), and MBP (p < 0.05) showed a significant variation among land-use types (Table 3). Furthermore, MBC, MBN, and MBP decreased by 70%, 76%, and 54% as a result of land use conversion from forest to cropland. Compared to the stoichiometric ratio of MBC:MBN, MBC:MBP, and MBN:MBP, a percentage decrease of 26%, 34%, and 46% from forest to cropland was observed.

**Table 5:** Descriptive statistics of microbial C, N, and P stoichiometry in land uses.

Variable	Land use	Min	Max	Mean	SD	CV (%)	Skewness	Kurtosis
MBC	Forest	174.82	239.13	202.77a	28.14	13.88	0.69	-0.68
	Savannah	98.10	157.47	130.74b	24.65	18.86	-0.69	1.37
	Grassland	51.01	110.31	87.45c	25.45	29.10	-1.45	2.71
	Fallow	46.98	59.69	53.91c	6.10	11.32	-0.26	-4.08
	Cropland	46.32	74.14	59.10c	12.46	21.08	0.37	-2.28
	All	83.45	128.15	106.79	19.36	18.85	-0.27	-0.59
MBN	Forest	15.85	19.61	17.66a	1.62	9.15	0.19	-1.03
	Savannah	11.59	13.99	12.91b	1.07	8.30	-0.46	-1.95
	Grassland	4.88	9.82	7.23c	2.36	32.59	0.14	-4.26
	Fallow	4.59	7.03	5.97cd	1.17	19.63	-0.38	-3.64
	Cropland	3.89	4.38	4.11d	0.21	5.00	0.63	0.42
	All	8.16	10.97	9.58	1.29	14.93	0.02	-2.09
MBP	Forest	5.94	8.93	7.20a	1.50	20.89	0.32	-4.18
	Savannah	5.91	7.38	6.73a	0.61	9.13	-0.73	1.36
	Grassland	1.99	9.43	6.89a	3.35	48.58	-1.72	3.10
	Fallow	2.07	5.79	4.13b	1.95	47.14	-0.14	-5.19
	Cropland	2.10	4.01	3.27b	0.89	27.10	-0.94	-0.75
	All	3.60	7.11	5.64	1.66	30.57	-0.64	-1.13

Different letters following mean values indicate a significant difference (P < 0.05) using a non-parametric multivariate comparison. Abbreviation: SD, Standard deviation; CV, Cumulative variance; MBC, Microbial biomass carbon; MBN, Microbial biomass nitrogen; MBP, microbial biomass phosphorus.

The ratio of MBC:MBN was statistically significant at p < 0.05. However, no significant difference was observed between MBC:MBP (p = 0.503) and MBN:MBP (p = 0.543) ratios across land-use types (Tables 6 and 7). Low MBN:MBP ratios were observed within land-use types. Ratios of MBN:MBP was not statistically significant (p > 0.05) and ranged between 1.18 and 2.89 with a mean of 1.85 across land-use types. The estimated values for microbial biomass values for C:N:P ratio were Forest = 11.49, 28.59, 2.52; Savannah = 10.12, 19.81, 1.94; Grassland = 12.34, 19.49, 1.69; Fallow = 9.23, 16.81, 1.76; and Cropland = 14.49, 18.67, 1.36. Also, SMB ratios for MBC:MBN, MBC:MBP and MBN:MBP were 11.53±2.22, 20.55±8.27, 1.85±0.80, respectively. This implies that the soils of Nkoranza (north and south) district were well-constrained. Hence, a soil microbial C:N:P ratio of 21:2:1 was recorded for the Nkoranza (north and south) district.

**Table 6:** Analysis of variance of microbial C, N, and P stoichiometry.

Source	DF	MBC	MBN	MBP	MBC:MBN Ratio	MBC:MBP Ratio	MBN:MBP Ratio
**1-Way ANOVA**							
Land use	4	0.001**	0.001**	0.031*	0.054*	0.503	0.543
Error		15.00	0.974	1.156	1.757	1.618	0.545

Values represent F values.

*p(F) < 0.05.

Abbreviation: MBC, Microbial biomass carbon; MBN, Microbial biomass nitrogen; MBP, Microbial biomass phosphorus; MBC:MBN Ratio, Microbial biomass carbon and Microbial biomass nitrogen ratio; MBN:MBP Ratio, Microbial biomass nitrogen and Microbial biomass phosphorus ratio; MBC:MBP Ratio, Microbial biomass carbon and Microbial biomass phosphorus ratio.

**Table 7:** Descriptive statistics of microbial C, N, and P stoichiometry in land uses.

Variable	Land use	Min	Max	Mean	SD	CV (%)	Skewness	Kurtosis
MBC:MBN Ratio	Forest	9.60	12.33	11.49ab	1.28	11.11	-1.85	3.46
	Savannah	7.83	11.26	10.12b	1.61	15.89	-1.45	1.69
	Grassland	10.45	16.65	12.34ab	2.89	23.45	1.92	3.76
	Fallow	7.38	11.03	9.23b	1.69	18.27	-0.06	-3.61
	Cropland	10.58	19.05	14.49a	3.63	25.07	0.44	0.44
	All	9.17	14.06	11.53	2.22	18.76	-0.20	1.15
MBC:MBP Ratio	Forest	23.46	31.57	28.59a	3.54	12.39	-1.59	2.93
	Savannah	13.28	26.64	19.81a	5.50	27.75	0.16	1.10
	Grassland	6.00	47.60	19.49a	19.05	97.72	1.81	3.35
	Fallow	8.11	28.84	16.18a	9.39	58.04	1.03	-0.12
	Cropland	13.31	22.10	18.67a	3.88	20.75	-1.20	-1.20
	All	12.83	31.35	20.55	8.27	43.33	0.04	1.21
MBN:MBP Ratio	Forest	1.90	3.07	2.52a	0.48	19.22	-0.42	0.68
	Savannah	1.68	2.37	1.94a	0.32	16.72	0.91	-1.00
	Grassland	0.57	4.32	1.69a	1.78	105.17	1.81	3.25
	Fallow	0.79	2.61	1.76a	0.89	50.61	-0.14	-4.63
	Cropland	0.97	2.09	1.36a	0.51	37.74	1.46	1.46
	All	1.18	2.89	1.85	0.80	45.89	0.72	-0.05

Different letters following mean values indicate a significant difference (P < 0.05) among the five land use types using a non-parametric multivariate comparison. Abbreviation: SD, Standard deviation; CV, Cumulative variance; MBC:MBN Ratio, Microbial biomass carbon and Microbial biomass nitrogen ratio; MBN:MBP Ratio, Microbial biomass nitrogen and Microbial biomass phosphorus ratio; MBC:MBP Ratio, Microbial biomass carbon and Microbial biomass phosphorus ratio.

### Relationship among soil and microbial C, N, and P stoichiometry in land uses

Fig 4 illustrates the degree of correlation between soil properties and their stoichiometric ratios. Pearson correlation values > 0.70 indicated that most of the indicators positively and negatively correlate with each other. Soil organic carbon (C) positively correlated with MBC (r = 0.75, p < 0.01) and MBN (0.69, p < 0.01). Also, nitrogen positively correlated with MBC (r = 0.59, p < 0.01), MBN (0.56, p < 0.05), and C (0.75, p < 0.01). Ratio of MBN:MBP correlated with MBC:MBP (r = 0.93, p < 0.01). Soil pH negatively correlated with MBP and C:N ratio at -0.50, respectively. Soil N positively correlated with MBN (r = 0.56; p < 0.05), and C (r = 0.69; p < 0.01).

Linear regression (relationship) indicated that R^2^ values of N and C were in the order: Forest (R^2^ = 0.9554), Savannah (R^2^ = 0.9132), Grassland (R^2^ = 0.8906), Fallow (R^2^ = 0.7659), and Cropland (R^2^ = 0.7650) (S1 Table; Fig 4). For N and P, there was no significant difference among the five land-use types. Also, there was no significant difference between C:N ratio and C for Forest (R^2^ = 0.3175), Savannah (R^2^ = 0.1721), Fallow (R^2^ = 0.3152), and Cropland (R^2^ = 0.0557) except Grassland (R^2^ = 0.7391). Also, no significant relationship between C:N ratio and N (R^2^ = 0.1433, 0.0274, 0.4241, 0.0104, and 0.0525) for Forest, Savannah, Grassland, Fallow, and Cropland, respectively. The relationship between C:P ratio and C indicated no significant linear correlation in Forest (R^2^ = 0.0363), Grassland (R^2^ = 0.0255), Fallow (R^2^ = 0.0492), and Cropland (R^2^ = 0.1935) except Savannah land use with a significant R^2^ value of 0.6120. However, no significant relationship was observed in C:P ratio and P (S1 Table). Also, N:P ratio had a positive significant linear correlation with N for forest (R^2^ = 0.6376) and Savannah (R^2^ = 0.5757), respectively. However, no significant correlation was observed for Grassland, Fallow, and Cropland (S1 Table). There was no significant correlation between N:P ratio and P (S1 Table and Fig 4). A similar trend was observed among microbial biomass MBC and MBN, MBC and MBP, MBC:MBN ratio and MBC, MBC:MBN ratio and MBP, MBN:MBP ratio and MBN, and MBN:MBP ratio and MBP, respectively (S2 Table). Soil C in Savannah had a weak positive relationship with MBC:MBP ratio (y = 7.2105x + 12.027, R^2^ = 0.2237, p < 0.05) than in Forest (y = 0.24x + 6.712, R^2^ = 0.0180, p < 0.05) and Cropland (y = 1.190x + 1.8388, R^2^ = 0.0181, p < 0.05) (S3 Table).

Furthermore, hierarchical cluster analysis with standardized data of soil and microbial C, N, and P and associated stoichiometric ratios of soil and microbial (C:N, C:P, N:P) were computed. At a distance > 20, cluster dendrograms were established using hierarchical clustering as illustrated in Figs 5 and 6. Fig 5 illustrates hierarchical clusters for the five land-use types. At a distance > 20, sampling sites exhibited three distinct cluster patterns. Cluster 1 consists of Forest, Cluster 2 (Savannah and Cropland), and Cluster 3 consists of 2 sub clusters. These are sub-cluster 1 (Savannah and Grassland) and sub-cluster 2 (Grassland and Fallow). At a distance greater than 50, sampling sites exhibited a cluster pattern of two major clusters. From Fig 6, two major clusters were identified and these clusters showed a significant influence on the contents and stoichiometric ratios of soil and microbial C, N and P. Cluster 1 consists of MBC and MBN and these two influenced the fertility and biological components of the soil. Cluster 2 comprised MBC:MBP, MBN:MBP and C:N ratios. For Cluster 3, concentration of soil (C, N, sub-cluster 1), and ratios (MBC:MBN, sub-cluster 2), C:P, and N:P (sub-cluster 3) were selected. N and P were the main indicators influencing soil nutrient concentration and microbial stoichiometric ratios.

## Discussion

### Effect of land use on soil C, N, and P stoichiometry

#### In soil nutrient concentration

A pH of 6.15 (Table 2) promotes the activities of microbes in tropical soils. Thus, highly productive ecosystems developed on acidic soils decrease P availability to plants and microbes in the warm and wet ecosystems of the tropics [76, 77]. An increase in the quantity of microorganisms promoted carbon sequestration and nitrogen fixation in Forest, Grassland and Savannah land-use systems. This improved soil physical conditions. Zhang et al. [78] observed that the prevailing soil microbes in Forest and Grasslands convert CO_2_ in the atmosphere into carbohydrates and biological nitrogen fixation may have enhanced nitrogen content and accumulation. For C concentration, the soil surface layer (0–20 cm) is the most biologically active. Hence, litter on the surface underneath the Forest canopy layers increased biomass production. This affirms the fact that the quality and quantity of litter is the most important factor influencing C concentration in the soil. These results were expected because most soil properties are controlled by organic matter content, which is consistent with studies of Takoutsing et al. [79] and Arnhold et al. [80]. These authors assessed the effect of land use on soil and microbial variables and observed that the highest accumulation of decayed and partially decomposed plants and animal residues was in the surface layers of the forest. This increases the interception and infiltration of water with little or no soil erosion [81]. Also, high clay content may have accounted for the differences in soil and microbial values of forests compared to other land-use systems.

The above explanation reaffirms that the micro-climate in the forest altered net primary productivity hence causing a considerable change to the quality and quantity of plant residues (litter and rhizo-deposition) resulting in increased soil nutrient levels [82–86]. This is because clay has a high capacity to adsorb organic matter and other soil nutrients. According to Van Veen and Kuikman [87] and Wardle [88], these reduce organic matter decomposition rate, buffer changes in soil pH, and provide soil microbes shelter from microbivores, thereby increasing soil water holding capacity. For cropland, the low soil nutrient levels may be attributed to the removal of large amounts of soil organic matter through harvesting, erosion, rapid decomposition processes, and disturbances from farm implements due to frequent and long-term tillage operations [89, 90]. Different soil layers are combined during tillage, resulting in the rapid decomposition of organic carbon [91] in Cropland. Also, C concentration in tropical soils decreases with increasing temperatures as a result this accelerates the decomposition of soil organic matter. This reduces the contribution of organic and microbial processes to soil nutrient cycling [92].

Total nitrogen in Forest was significantly different from the other land-use systems (p < 0.05; Table 3). Relatively, organic matter is the main source of nitrogen in most soils. Forest had the highest total nitrogen due to the high organic matter content. Also, the presence of Azotobacter algae can fix atmospheric nitrogen from decaying plants and animal matter and through nitrogen compounds produced from thunderstorms in the tropics [93]. Another possible reason is related to the composition of rhizobiales, a branch of Alpha-proteobacteria class considered as rhizospheric plant-promoting bacteria [94, 95] that can fix atmospheric nitrogen (N_2_) in symbiosis with plants [96]. This has resulted in increasing proteo-bacteria and its abundance can contribute to nitrogen accumulation hence affecting the N:P ratio. The mean value of N (0.09%) under cropland was adequate compared to other land-use systems [89], Table 2). This implies that fertiliser application generally stimulates plant growth and carbon input [97] and this did replace N lost due to harvest removal, leaching and humus losses associated with cultivation [90, 98]. Also, the contribution of mineral fertiliser, farmyard manure, and organic matter from which total nitrogen is mainly derived for crop production was high on cropland. However, to maintain these levels, site-specific application of fertiliser (urea, various forms of ammonium or nitrate salts such as ammonium nitrate, potassium nitrate, etc.) directly should be encouraged to increase the yield on croplands.

Also, phosphorus content for Forest and Grassland was high as compared to Cropland. This is because phosphorus was captured in deeper layers by plant roots and deposited on the surface as organic residues when these leaves and branches fall on the soil surface [99, 100]. In general, phosphorus levels were low in land use types, except Forest soils [54, 101]. The inherently low phosphorus level is due to the low concentration of phosphorus in the parent material forming these soils (Table 3). Hence, tropical soils low in phosphorus concentration [102] affect P—fixation [103] According to Ayodele and Omotoso [104], available phosphorus of 8.5 mg kg^-1^ is below the critical level required by most crops. Some plants require P > 8 mg kg^-1^, while moderate-demanding crops > 14 mg kg^-1^ and high—demanding crops require P > 21 mg kg^-1^. Again, the low phosphorus values may be due to the acidic nature (pH = 5.88 for Cropland; Table 2) of the soils, which falls outside the optimal range of 6.5–7.0. Also, its deficiency may be attributed to anthropogenic perturbation (eg. deforestation, overgrazing, over-cultivation, erosion etc. [61, 105]).

Our results indicated that Cropland had low phosphorus compared to Fallow and other land-use systems. This is attributed to low fertiliser application [106]. The low application is due to the high cost of fertilisers and the low purchasing power of these smallholder farmers in the Nkoranza district. Therefore, the maize fields (Cropland) sampled can be supplied with inorganic P-fertilisers to increase P concentration in the soil solution to meet crop requirements. Phosphorus in fertiliser added to the soil considerably increases the concentration of phosphorus available for crop uptake. However, when phosphorus is applied in excess, it precipitates out of the soil through chemical reactions with charged molecules. Bewket and Stroosnijder [107] and Yifru and Taye [91] reported a similar trend in their studies. Also, most of the nutrient loss on these small farms contains more than three times the nutrients left behind after erosion, and it is 1.5–5 times rich in SOM [108, 109]. Thus, in managed systems (cropland) nutrient addition is mostly practiced whilst in natural systems (Forest) nutrient deposition dominates [86].

#### In stoichiometric ratios

Furthermore, stoichiometric ratios of C:N, C:P, and N:P were used as indicators of soil fertility [100]. Soil C:N ratio is a valuable indicator of organic matter decomposition, and its potential contribution to soil fertility [110, 111]. According to Hazelton and Murphy [112] and Fazhu et al. [113], a high C:N (> 25) indicates a high SOM accumulation compared to decomposition. Further, the 0–20 cm soil layer had a higher C:N ratio (13.91) because a more active carbon had been sequestered. This indicates the complete breakdown of organic matter in the soils [114]. Also, the release of carbon and other nutrients results in microbial decomposition and accumulation of litter and fine plant roots in the soil medium. The turnover rates of these fine roots are very high, resulting in the build-up of organic carbon as inputs to the soil [114–116].

Additionally, C:P ratio is considered an indicator of high phosphorus availability. The low C:P ratio recorded indicates net phosphorus mineralisation [117] (Table 4). For C:P ratio, our results ranged from 0.23 to 0.52, with a mean of 0.88 indicating net phosphorus mineralisation. In a similar research by Paul [117], a C:P ratio < 200 means net mineralisation. Also, C:P ratio > 300 refers to net immobilization, and a C:P ratio value between 200 and 300 implies a slight change in the concentration of soil-soluble phosphorus. The N:P ratio is accepted as an indicator of nutrient limitation and its decomposability [30]. According to Wardle et al. [88], an N:P ratio > 16 indicates soil P limitation in ecosystems. From Table 4, N:P ratio ranged between 0.01 and 0.19 with a mean value of 0.06 indicating high soil microbial activity. Also, N:P ratio is accepted as an indicator of nutrient limitation and its decomposability [29]. According to Wang et. al. [118] an N:P ratio > 2 shows a decline in biomass. Our results revealed a low value of 0.06 for all land use indicates an increase in biomass production. The N:P ratio, also regarded as an indicator of N-saturation is used to determine soil nutrient limitation thresholds. The N:P ratio is regarded as a diagnostic value because this ratio is improved during soil-plant fertilisation processes [119].

Also, low C:P and N:P ratios were observed due to the low availability of phosphorus, derived primarily from rock weathering [120]. Therefore, a decline in these could be due to a decrease in phosphorus due to an increase in its demand by plants as a result of biomass accumulation [115, 116, 120] in Grassland, Savannah woodland, and Forest land-use systems. In highly weathered tropical soils, phosphorus limitation is due to a decline in phosphorus content in plant litter. This implies that less phosphorus is returned to the soil through plant decomposition. Also, the release of C and other nutrients results in microbial decomposition and accumulation of litter and fine plant roots in the soil medium. The turnover rates of these fine roots are very high, resulting in the build-up of C as inputs to the soil [114–116]. This implies that a large amount of fine root biomass and their turnover (dead roots) increased in Forest, Savannah woodland, and Grassland resulting in an increase in C and N contents [28, 116, 121].

Also, low phosphorus concentration resulted in a low C:P ratio and N:P ratio. A low C:P ratio indicates a high soil P availability. Therefore, the results of this study showed that soil P availability was relatively high and that the mineralisation rate of P was relatively high. Soil N:P ratio values served as an indicator of N saturation. This was used to determine the nutrient limitation thresholds of the study area. However, an N:P ratio < 10 is an indication of N-limitation. This implies that an N:P ratio value of 0.06 is low, which explains the low levels of N in the study area.

In a similar research, Metwally et al. [114] and Ouyang et al. [122] had low correlation values for C:N ratio, C:P ratio and N:P ratio, which emphasised the importance of P and N limitation to primary ecosystem production processes in tropical soils. This has resulted in N and P being the most limiting element in tropical agroecosystems. The limitation of phosphorus in the study area is because phosphorus primarily is derived from weathering of the parent rock (thus low phosphorus parent materials: low inputs of phosphorus via weathering due to the low concentrations of phosphorus in rock). This implies that the weathered old soils of the study sites are depleted of phosphorus and phosphorus depletion resulted in biological phosphorus limitation [120].

Furthermore, the mechanisms that influence phosphorus limitation include the depletion of phosphorus through anthropogenic sources. This results in the formation of soil layers that physically prevent and/or inhibit access by plant roots to potentially available phosphorus sources. This resulted in the separation of biota from phosphorus and phosphorus-bearing minerals (soil barrier-driven P-limitation). Also, low-P parent material forming soils caused the constraints and limitations of phosphorus [120]. According to Vitousek [123], Chadwick et al. [124], and Hedin et al. [125], 90% of soil phosphorus from parent material is lost through the leaching of dissolved organic P.

Our findings indicated that pedogenic soil barriers prevalent in the study area influenced P availability and limitation. The formation of iron pans in high rainfall areas of the study area may have constrained root access to deeper layers, thereby restricting drainage. As a result, water is rerouted horizontally than vertically into the soil. This affects phosphorus supply compared to N, hence making phosphorus limitation more pronounced in the Nkoranza district. Also, N can be constrained by bushfires prevalent in the study area. The activities of fire may have volatilised N compared to phosphorus. These human-induced disturbances play a major development in N compared to P limitation. The mobility of N implies that N losses can be substantial whenever available N supply exceeds demand. In a study by Davidson et al. [126] in Amazon ecosystems, N losses associated with human disturbances to P-limited biomes altered N:P stoichiometry to induce an N limitation in forest and other land-use systems. These findings by Davidson et al. [126], for N:P ratio and MBN:MBP ratio were consistent with our N:P ratio which was highly constrained. From the above discussion, it can be concluded that N and P limitation plays an essential role in soil nutrient dynamics.

### Effect of land use on microbial C, N and P stoichiometry

Soil microbial biomass plays an essential role in soil fertility determination. An essential function is to accumulate and conserve soil nutrients in a biologically active form during high biomass and low turnover (dry season). When the activities of plants are low they cannot extract nutrients effectively from the soil (low biomass and high turnover wet season) to increase plant growth and development. Also, microbial biomass indicates C, N, and P retention, and a reduction in biomass results in the mineralisation of nutrients and an increased result in soil nutrient retention [127, 128]. Forest had the highest MBC, MBN and MBP due to the accumulation of litter on the surface (Table 5).

**Fig 3:**
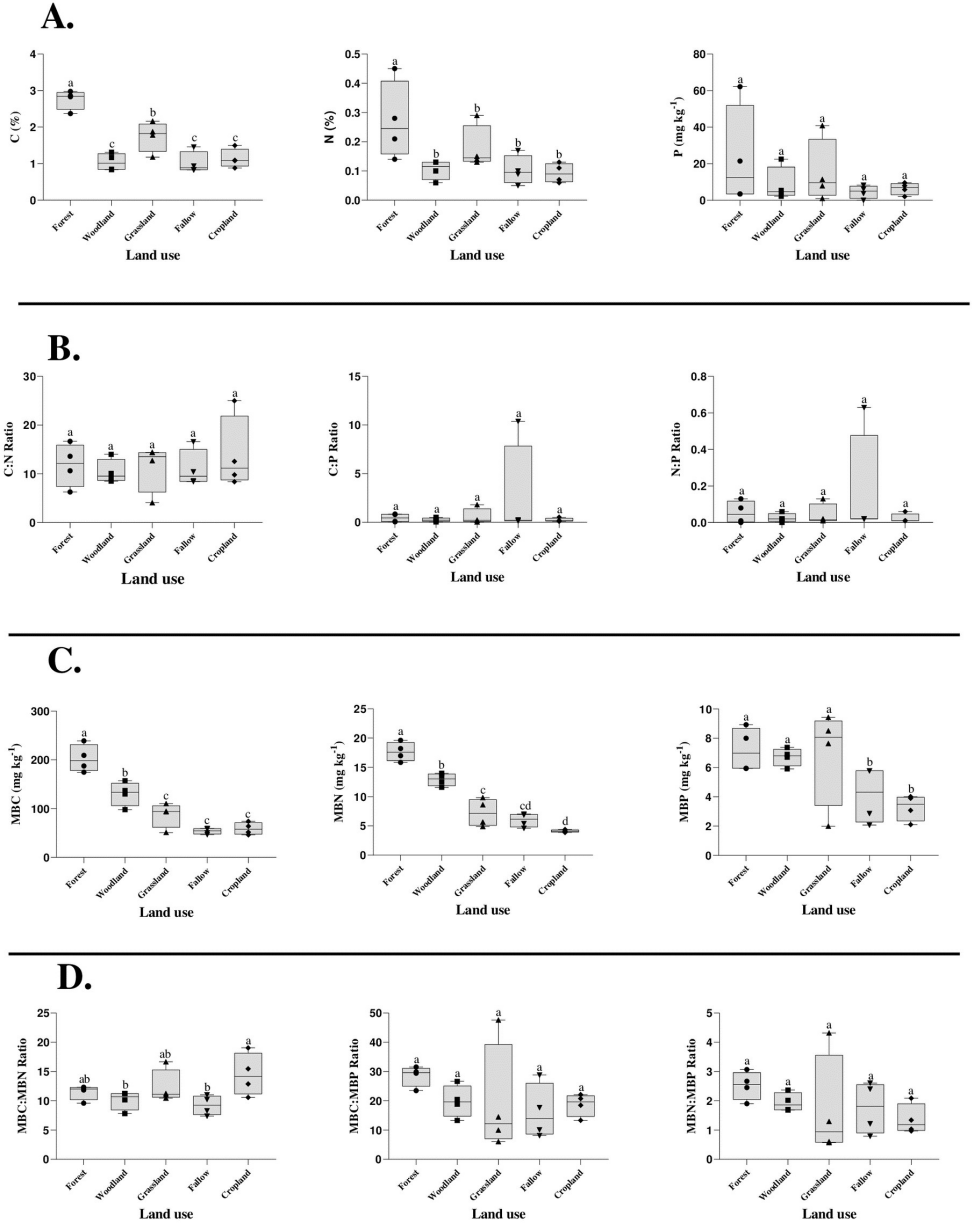
Box and whisker plots indicating stoichiometric differences in soil and microbial C, N, and P with outliers. On each whisker, letters represent significant differences at p < 0.05. Box-plots assigned the same letters are not statistically different (p > 0.5, Duncan’s test).

The constant supply of litter from falling leaves, dead branches, and decomposing roots favour the growth of soil microbes, resulting in the accumulation of MBC [129]. Among land-use types, Forests had relatively high ratios of MBC:MBP and MBN:MBP (Table 7) due to leguminous plants, and N_2_-fixing plants may have contributed to soil nutrient cycling in the Forest. On the other hand, cropland had low MBC, MBN, and MBP compared to different land-use types. According to Wright and Reddy [130], soil nutrient availability influences soil microbial activity and function. A weak positive correlation between available phosphorus and MBP was observed (Fig 3). This indicates a decline in available phosphorus due to an increase in soil acidity and microbial activity in land-use systems [131].

MBC and MBN of the Forest were high due to the addition of stable organic matter inputs in the soil. Soils of the natural forest have numerous roots with large aboveground biomass. This provided abundant litter for microorganisms [132]. This explains the high microbial biomass concentration in forest and savannah woodland [35]. The forest provided a suitable environment for phyla enabling them to utilize the degradable fractions of plant residues and this facilitated carbon accumulation [133] effect on the C:N:P stoichiometry [134]. In grassland, MBC and MBN were affected by litter decomposition through their numerous root exudates. As a result, grassland accumulated a substantial amount of above-ground biomass. This serves as a nutrient that fuel microbial activities and increased the production of MBC and MBN. Also, the above-ground biomass of grassland is grazed by livestock. Soil microorganisms provide nutrients for plants through the decomposition of SOM. This implies that soil MBC is closely related to carbon storage, which is consistent with Li et al. [6]. From the above, microbial biomass carbon, nitrogen, and phosphorus ratios had significant stability with ratios of 106.79 (p < 0.001), 9.58 (p < 0.001), and 5.64 (p < 0.031) (Table 5 and 6). Ratios of MBC:MBN, MBC:MBP and MBN:MBP indicated that the C-N-P in different land-use types possess homeostatic characteristics. Also, Marklein and Houlton [135] observed that microbes remain relatively homeostatic concerning C:P, and N:P ratios under P-limited conditions.

### Relationships between soil and microbial C, N, and P stoichiometry

**Fig 4:**
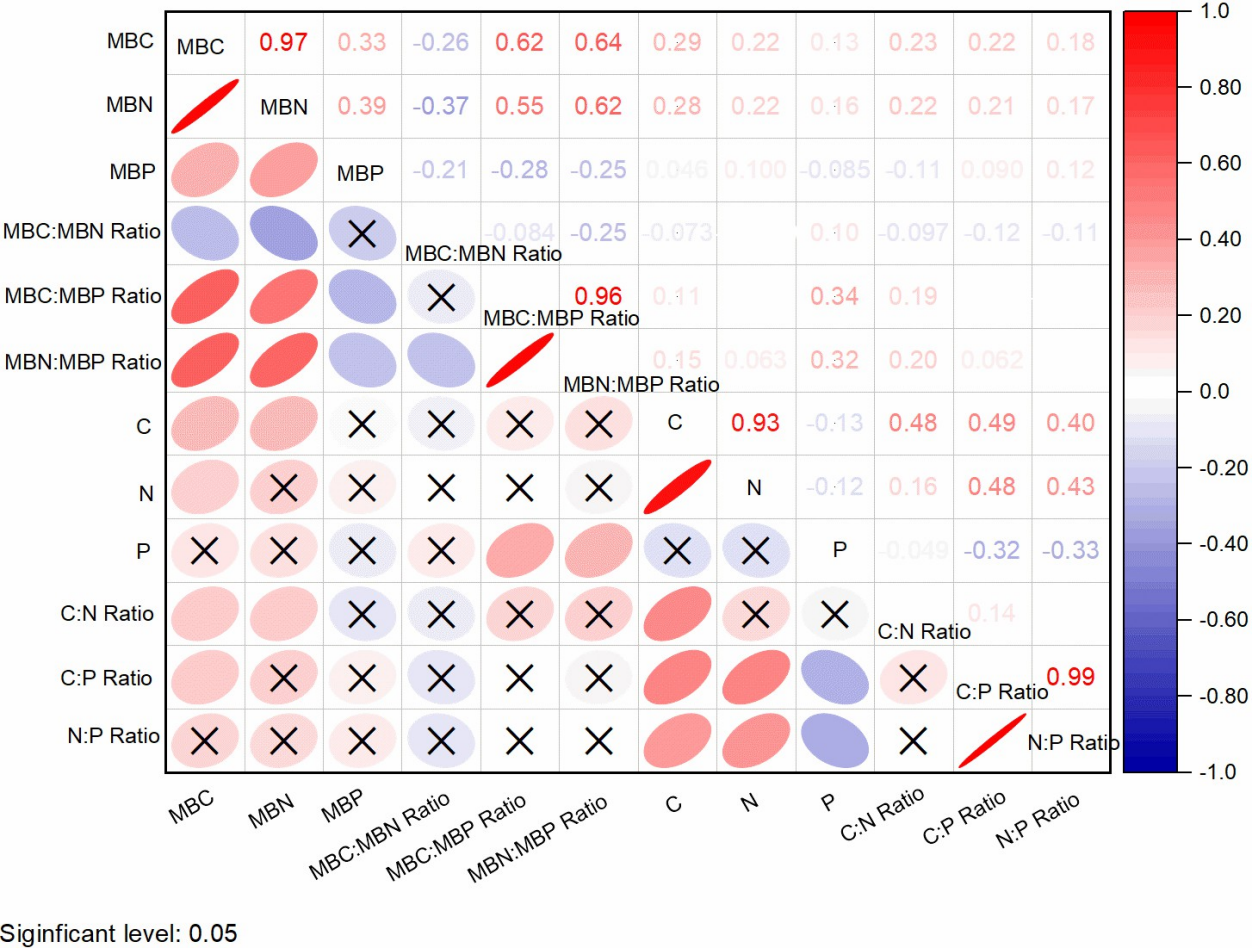
Correlation coefficients (r) of soil and microbial biomass C, N, and P concentration and their stoichiometric ratios.

A high correlation coefficient between C and N concentrations (r = 0.69; p < 0.01; Fig 4) was observed, indicating a highly constrained C:N ratio in this study. Also, there were relatively constrained C:P and N:P ratios with correlation coefficients for C and P concentrations at 0.31 and N and P concentrations at 0.23 (p < 0.05). This implies a relatively constrained C:N:P ratio of soils and these findings were consistent with several studies [28, 100, 115, 136]. From Fig 4, MBC, MBN, and MBP significantly correlated positively with C, N, and P (p < 0.01, p < 0.05). This means that MBC, MBN, and MBP are closely related to C, N, and P contents [129, 137]. These authors concluded that nitrogen dynamics in soil are associated with C and MBC, and this revealed a significant positive correlation (0.75; p < 0.01; Fig 4) with MBN. This means N increased with an increase in C concentration. Hence, MBC, MBN, and MBP contents as well as organic matter are important factors that determine microbial biomass carbon and nitrogen accumulation in soils of the study area.

Further, a strong positive correlation was observed between ratios of MBN:MBP and MBC:MBP (r = 0.96). Also, ratios of MBC:MBN and MBC:MBP related to SOM can serve as an indicator of organic matter and nitrogen supply. This implies that low MBC:MBN and MBC:MBP ratios indicate a high nitrogen and phosphorus availability. According to Hackl et al. [138], several factors stressed the effects of land-use type on soil microbial biomass. The difference in the quality and quantity of substrate input through different litter and plant root types with their associated nutrient specificity serves as drivers that influence soil microbial biomass [100] in agroecosystems. A change in land use altered the stoichiometric ratios of carbon, nitrogen, and available phosphorus. A significant reduction in phosphorus due to its low availability in these highly weathered tropical soils may have resulted in low C:P and N:P ratios in the study area (Table 4). From the above, a change in land use from Forest to Cropland decreased soil carbon, nitrogen and phosphorus, and this reduced soil microbial biomass activity in the soil medium.

**Fig 5:**
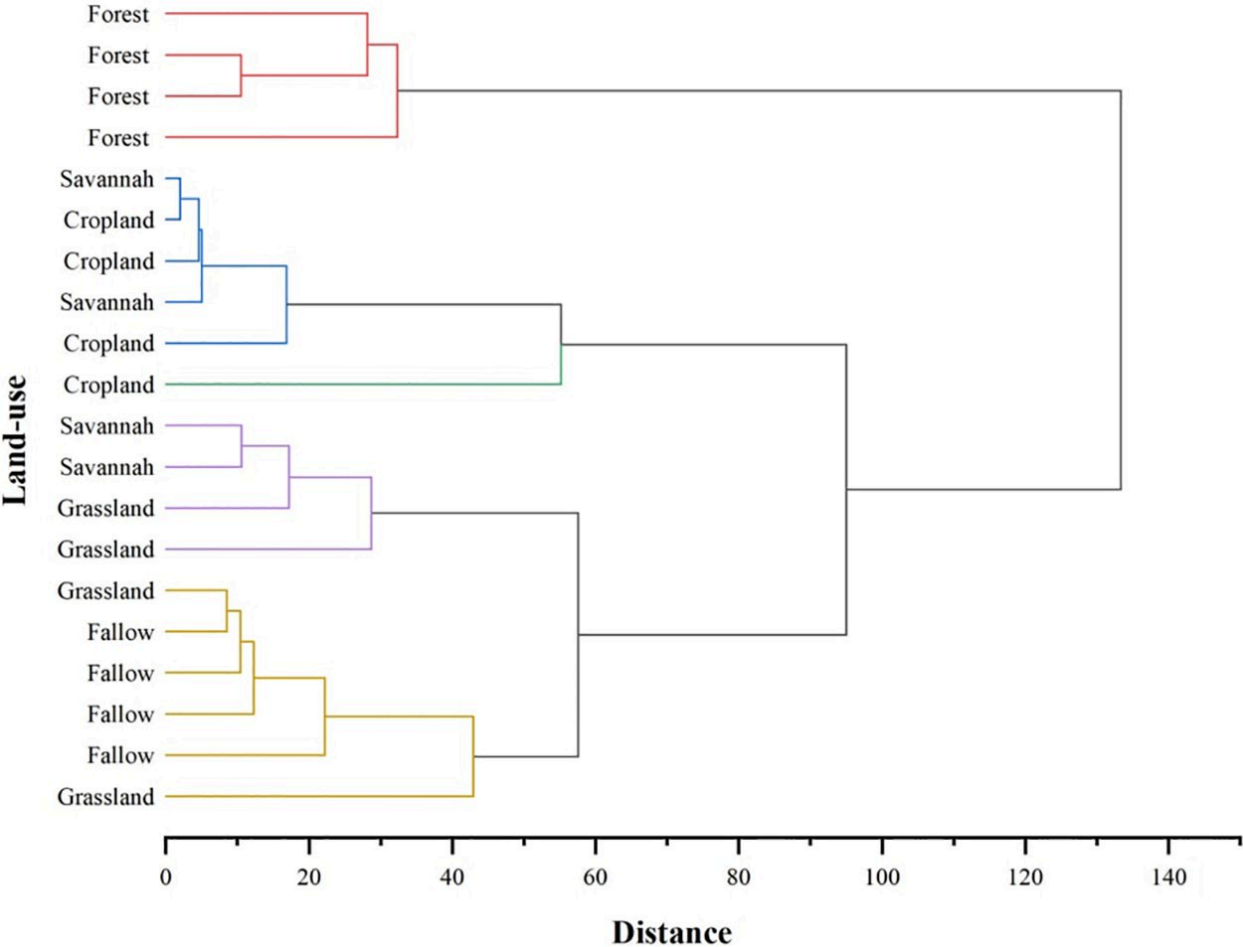
A hierarchical clustering of soil C, N, and P stoichiometry in land uses of the study area.

**Fig 6:**
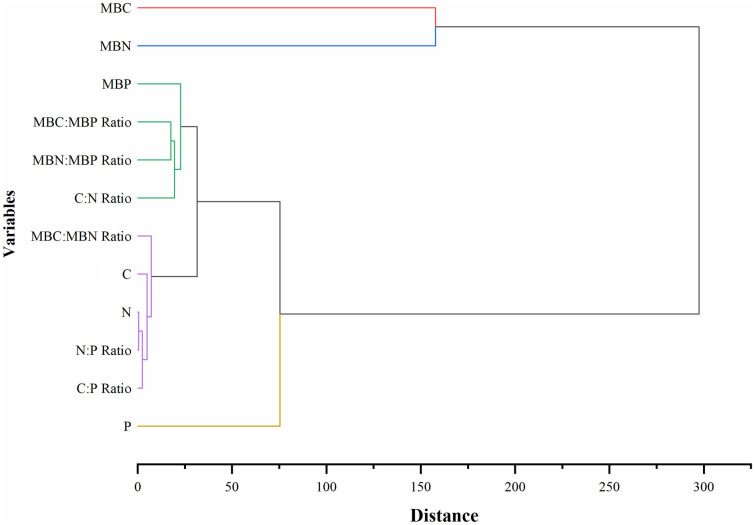
A hierarchical cluster of soil C, N, and P stoichiometry in soils of the study area.

Additionally, cluster dendrograms were established using hierarchical clustering as illustrated in Figs 5 and 6. At a distance greater than 20 (Fig 5), sampling sites exhibited three distinct clusters. These three distinct clusters reaffirm the fact that land-use types were significantly affected due to the conversion of Forest to Cropland and other land uses (Fig 6; Tables 2 and 4–6). At a distance greater than 50 (Fig 6), sampling sites exhibited a significant influence on the contents and stoichiometric ratios of soil and microbial C, N, and P. Cluster 1 consisted of MBC and MBN. These two indicators influenced the fertility and biological components of soils in the study area. Cluster 2 comprised MBC:MBP, MBN:MBP and C:N ratios. Ratios of microbial C:P and N:P were generally low in the study area indicating that soil organic matter decomposes rapidly and would be useful to compensate for the decomposed organic matter through appropriate agricultural practices. Hence, soil C:N ratio, an indicator of soil nutrient balance influenced the sensitivity of land-use types to anthropogenic perturbation. Microbial C:P ratio as an indicator assisted in the determination of phosphorus concentration released from the mineralized soil. For Cluster 3 (N:P and C:P of sub-cluster 3), phosphorus constituting the major limiting nutrient factor was single-handedly selected. This confirms the assertion that phosphorus was the most limiting nutrient in the Nkoranza (north and south) district in the Forest-Savannah Transition Zone of Ghana as well as in tropical soils.

## Conclusion

We conclude that C:N:P stoichiometry is an important indicator of elemental balance in the study of ecological interactions and processes. Land use significantly influenced soil and microbial biomass C, N, and P and its stoichiometry in these highly weathered tropical nutrient-poor systems. Native forest had the highest stochiometric ratio in soil microbial biomass C, N, and P compared to cropland. The estimated C:N:P ratios, known as the “Redfield ratio” for microbial biomass (21:2:1) were well constrained (homeostasis) in the study area. Also, a significant relationship (p < 0.01) observed between soil nutrients and microbial biomass facilitates the use of organic matter as an essential indicator for soil fertility assessment in agricultural production systems. The transition from Forest to Cropland resulted in 64%, 52%, and 71% reduction in C, N, and P respectively. This implies that phosphorus is the main factor limiting productivity. Thus, a significant reduction in phosphorus due to its low availability in these highly weathered tropical soils may have resulted in low C:P and N:P ratios in the Nkoranza (north and south) district.

From the above discussion, we can conclude that the changing intensity in C, N, and P was influenced by the intrinsic characteristics of these highly weathered soils inherently low in phosphorus. These results suggest that the soil management interventions (such as agroforestry, cover cropping, mixed cropping, and adoption of integrated soil fertility management (ISFM) principles thus the use of organic and inorganic fertilizers, composting, and/or mulching) should be encouraged. Also, afforestation has the potential to increase below-ground microbial composition and this alteration can affect organic matter decomposition in biogeochemical cycles. These principles should be based on land use and site-specific information on soil nutrient management. These findings stress that a desirable change can intensify effective management strategies (ISFM principles) that seek to restore degraded farmlands. Therefore, we recommend increasing nutrient inputs in croplands in the Nkoranza district to improve soil fertility. Further, the effects of land use on biological and chemical characteristics and their relationship with nutrient status (soil and microbial C, N, and P) at 1-meter depth will be considered in our future works in the Forest-Savannah Transition Zone of Ghana.

## Supporting information

S1 Table Illustrates linear regression analysis of C, N and phosphorus and stoichiometry ratios of different land use systems. Abbreviation: pH, soil pH; C, Soil organic carbon; N, Total nitrogen; phosphorus, Available phosphorus; C:N Ratio, Carbon:nitrogen ratio; C:P Ratio, Carbon and phosphorus ratio; N:P Ratio, Nitrogen and phosphorus Ratio.
(PDF)

S2 Table Linear regression analysis of MBC, MBN and MBP stoichiometry ratios of different land use systems. Abbreviation: MBC, Microbial biomass carbon; MBN, Microbial biomass nitrogen; MBP, Microbial biomass phosphorus; MBC:MBN, Microbial biomass carbon and nitrogen ratio; MBC:MBP, Microbial biomass carbon and phosphorus ratio; MBN:MBP, Microbial biomass nitrogen and phosphorus.
(PDF)

S3 Table Illustrates linear regression analysis of soil(C), microbial biomass (MBC, MBN, MBP), and stoichiometric ratios (MBC:MBN, MBC:MBP, MBN:MBP) of different land use systems. Abbreviation: C, Soil organic carbon; MBC, Microbial biomass carbon; MBN, Microbial biomass nitrogen; MBP, Microbial biomass phosphorus; MBC:MBN Ratio, Microbial biomass carbon and Microbial biomass nitrogen ratio; MBN:MBP Ratio, Microbial biomass nitrogen and Microbial biomass phosphorus ratio; MBC:MBP Ratio, Microbial biomass carbon and Microbial biomass phosphorus ratio.
(PDF)
