# Supplementary material for: Human-induced land use changes and phosphorus limitation affect soil microbial biomass and ecosystem stoichiometry
Source: PLoS One. 2023 Aug 30;18(8):e0290687. doi: 10.1371/journal.pone.0290687 (PMC10468095; doi:10.1371/journal.pone.0290687)
Supplement: S1 Table — Abbreviation: pH, soil pH; C, Soil organic carbon; N, Total nitrogen; phosphorus, Available phosphorus; C:N Ratio, Carbon:nitrogen ratio; C:P Ratio, Carbon and phosphorus ratio; N:P Ratio, Nitrogen and phosphorus Ratio. (PDF) [file pone.0290687.s001.pdf]

S1 Table. Illustrates Linear regression analysis of C, N and P and Stoichiometry ratios of different land use systems.

X	Land use	Y	
		C	P
N	Forest	y = 9.1630x - 0.0746	y = -122.9000x + 32.5000
		R^2 = 0.9554	R^2 = 0.3138
	Savannah	y = 8.2420x - 0.0292	y = -42.6700x + 10.8200
		R^2 = 0.9132	R^2 = 0.1232
	Grassland	y = 12.5700x - 0.6210	y = 82.6400x – 3.8010
		R^2 = 0.8906	R^2 = 0.0659
	Fallow	y = 7.6080x - 0.0543	y = 77.4300x + 1.2810
		R^2 = 0.7659	R^2 = 0.0220
	Cropland	y = 7.0110x + 0.04424	y = -27.2400x + 12.1100
		R^2 = 0.7650	R^2 = 0.009607
X	**Land use**	Y	
		C	N
C:N Ratio	Forest	y = 0.3625x - 1.626	y = 0.0256x - 0.05368
		R^2 = 0.3175	R^2 = 0.1433
	Savannah	y = 0.1228x - 0.1704	y = 0.0057x + 0.05530
		R^2 = 0.1721	R^2 = 0.02744
	Grassland	y = 0.2833x - 1.107	y = 0.0161x + 0.0125
		R^2 = 0.7391	R^2 = 0.4241
	Fallow	y = 0.1597x - 0.3139	y = 0.0033x + 0.09123
		R^2 = 0.3152	R^2 = 0.01046
	Cropland	y = 0.0599x + 0.4193	y = -0.0073x + 0.1720
		R^2 = 0.0557	R^2 = 0.05250
X	**Land use**	Y	
		C	P
C:P Ratio	Forest	y = 0.0236x + 1.4010	y = -0.7619x + 13.8000
		R^2 = 0.03636	R^2 = 0.0691
	Savannah	y = 2.3370x + 0.3869	y = -23.6500x + 10.6800
		R^2 = 0.6120	R^2 = 0.3155
	Grassland	y = -0.1234x + 1.202	y = -7.1560x + 10.9000
		R^2 = 0.0255	R^2 = 0.1465
	Fallow	y = -0.3779x + 0.8975	y = -42.6500x + 18.8600
		R^2 = 0.04925	R^2 = 0.1739
	Cropland	y = 0.7288x + 0.7137	y = -23.4400x + 13.8900
		R^2 = 0.1935	R^2 = 0.1665
X	**Land use**	Y	
		N	P
N:P Ratio	Forest	y = 0.9145x + 0.1082	y = -124.7000x + 19.9900
		R^2 = 0.6376	R^2 = 0.2464
	Savannah	y = 1.9290x + 0.05728	y = -188.3000x + 10.7200
		R^2 = 0.5757	R^2 = 0.3710
	Grassland	y = -0.0315x + 0.1426	y = -53.9100x + 10.9400
		R^2 = 0.0057	R^2 = 0.1622
	Fallow	y = -0.6231x + 0.1317	y = -337.0000x + 19.2400
		R^2 = 0.1714	R^2 = 0.1839
	Cropland	y = 0.4660x + 0.1038	y = -174.9000x + 14.0900
		R^2 = 0.0910	R^2 = 0.1660

Abbreviation: pH, soil pH; C, Soil organic carbon; N, Total nitrogen; P, Available phosphorus; C:N ratio, Carbon:nitrogen ratio; C:P ratio, Carbon and phosphorus ratio; N: ratio, Nitrogen and phosphorus ratio.
