# Supplementary material for: Human-induced land use changes and phosphorus limitation affect soil microbial biomass and ecosystem stoichiometry
Source: PLoS One. 2023 Aug 30;18(8):e0290687. doi: 10.1371/journal.pone.0290687 (PMC10468095; doi:10.1371/journal.pone.0290687)
Supplement: S2 Table — Abbreviation: MBC, Microbial biomass carbon; MBN, Microbial biomass nitrogen; MBP, Microbial biomass phosphorus; MBC:MBN, Microbial biomass carbon and nitrogen ratio; MBC:MBP, Microbial biomass carbon and phosphorus ratio; MBN:MBP, Microbial biomass nitrogen and phosphorus. (PDF) [file pone.0290687.s002.pdf]

S2 Table. Linear regression analysis of MBC, MBN and MBP Stoichiometry ratios of different land use systems

X	**Land use**	Y	
		MBN	MBP
MBC	Forest	y = 0.7417x – 4.2949	y = 0.1284x + 10.5900
		R^2 = 0.8722	R^2 = 0.0763
	Savannah	y = 0.6182x + 0.7319	y = -0.0545x + 17.3600
		R^2 = 0.9488	R^2 = 0.04805
	Grassland	y = 0.5595x + 6.7090	y = 0.0116x + 8.4470
		R^2 = 0.8357	R^2 = 0.0218
	Fallow	y = 0.8122x – 2.5810	y = 0.0630x + 10.2000
		R^2 = 0.7371	R^2 = 0.0097
	Cropland	y = 0.6948x - 0.8869	y = 0.1584x + 7.1610
		R^2 = 0.9471	R^2 = 0.0180
X	**Land use**	Y	
		MBC	MBN
MBC:MBN Ratio	Forest	y = -15.4600x + 157.1000	y = -75.2000x + 204.6000
		R^2 = 0.0043	R^2 = 0.1597
	Savannah	y = -30.5600x + 110.5000	y = -28.1500x + 84.8700
		R^2 = 0.0551	R^2 = 0.1160
	Grassland	y = 66.8700x - 37.0800	y = 11.8400x + 23.8900
		R^2 = 0.2819	R^2 = 0.0236
	Fallow	y = -27.4700x + 81.9600	y = -43.4700x + 94.9600
		R^2 = 0.1658	R^2 = 0.4637
	Cropland	y = -8.6210x + 29.3400	y = -7.9180x + 22.9000
		R^2 = 0.2459	R^2 = 0.4070
X	**Land use**	Y	
		MBC	MBP
MBC:MBP Ratio	Forest	y = 0.0419x + 2.1312	y = -3.3820x + 54.1900
		R^2 = 0.2147	R^2 = 0.4326
	Savannah	y = 0.0945x + 0.7839	y = -1.0080x + 20.5200
		R^2 = 0.7821	R^2 = 0.1875
	Grassland	y = 0.1122x + 0.1885	y = -0.2327x + 10.8400
		R^2 = 0.6654	R^2 = 0.1649
	Fallow	y = 0.1302x + 0.0041	y = -1.3380x + 20.1100
		R^2 = 0.3475	R^2 = 0.2139
	Cropland	y = -0.0439x + 3.4680	y = -1.3360x + 13.2000
		R^2 = 0.0126	R^2 = 0.1908
X	**Land use**	Y	
		MBN	MBP
MBN:MBP Ratio	Forest	y = 4.9260x + 69.4100	y = -4.3590x + 51.1000
		R^2 = 0.1568	R^2 = 0.3585
	Savannah	y = 8.4080x + 3.8980	y = -1.6570x + 20.6600
		R^2 = 0.7366	R^2 = 0.1864
	Grassland	y = 5.2100x + 16.1400	y = -0.3824x + 11.0300
		R^2 = 0.7290	R^2 = 0.2369
	Fallow	y = 3.4610x + 18.2600	y = -1.7990x + 19.6200
		R^2 = 0.2965	R^2 = 0.1757
	Cropland	y = 0.1395x + 8.6860	y = -2.0750x + 12.9600
		R^2 = 0.0021	R^2 = 0.1686

Abbreviation: MBC, Microbial biomass carbon; MBN, Microbial biomass nitrogen; MBP, Microbial biomass phosphorus; MBC:MBN Ratio, Microbial biomass carbon and Microbial biomass nitrogen ratio; MBN:MBP Ratio, Microbial biomass nitrogen and Microbial biomass phosphorus ratio; MBC:MBP Ratio, Microbial biomass carbon and Microbial biomass phosphorus ratio.
