# Supplementary material for: Human-induced land use changes and phosphorus limitation affect soil microbial biomass and ecosystem stoichiometry
Source: PLoS One. 2023 Aug 30;18(8):e0290687. doi: 10.1371/journal.pone.0290687 (PMC10468095; doi:10.1371/journal.pone.0290687)
Supplement: S3 Table — Abbreviation: C, Soil organic carbon; MBC, Microbial biomass carbon; MBN, Microbial biomass nitrogen; MBP, Microbial biomass phosphorus; MBC:MBN Ratio, Microbial biomass carbon and Microbial biomass nitrogen ratio; MBN:MBP Ratio, Microbial biomass nitrogen and Microbial biomass phosphorus ratio; MBC:MBP Ratio, Microbial biomass carbon and Microbial biomass phosphorus ratio. (PDF) [file pone.0290687.s003.pdf]

S3 Table. Illustrates linear regression analysis of soil (C), microbial biomass (MBC, MBN, MBP), and stoichiometry ratios (MBC:MBN, MBC:MBP, MBN:MBP) of different land use systems

X	**Land use**	Y		
		MBC	MBN	MBP
C	Forest	y = 12.306x + 116.78	y = 5.7313x + 87.275	y = 0.6796x + 29.796
		R^2 = 0.0283	R^2 = 0.0097	R^2 = 0.0004
	Savannah	y = -57.439 + 104.01	y = -32.08x + 62.308	y = -0.7302x +14.756
		R^2 = 0.1619	R^2 = 0.1254	R^2 = 0.0004
	Grassland	y = 15.551x + 44.24	y = 2.486x + 38.604	y = 1.2697x + 6.2104
		R^2 = 0.0555	R^2 = 0.0038	R^2 = 0.0241
	Fallow	y = 10.854x + 32.839	y = 18.022x + 16.535	y = -0.745x + 8.6289
		R^2 = 0.0233	R^2 = 0.0719	R^2 = 0.0014
	Cropland	y = 6.6234x + 8.3696	y = 4.9169x + 4.654	y = -4.2915x + 13.09
		R^2 = 0.085	R^2 = 0.0919	R^2 = 0.0248
X	**Land use**	Y		
		MBC:MBN Ratio	MBC:MBP Ratio	MBN:MBP Ratio
C	Forest	y = -0.0061x + 1.4598	y = 0.24x + 6.712	y = 0.2019x + 4.4771
		R^2 = 0.0004	R^2 = 0.0018	R^2 = 0.003
	Savannah	y = 0.361x + 1.4197	y = -7.2105x + 12.027	y = -4.0158x + 7.1061
		R^2 = 0.1085	R^2 = 0.2237	R^2 = 0.1887
	Grassland	y = 0.1735x + 1.2842	y = -0.6905x + 14.816	y = -1.5364x + 11.963
		R^2 = 0.1107	R^2 = 0.0003	R^2 = 0.0023
	Fallow	y = -0.0999x + 1.5462	y = -18.784x + 46.177	y = -11.064x + 31.177
		R^2 = 0.0089	R^2 = 0.0037	R^2 = 0.0026
	Cropland	y = -0.2077x + 1.9448	y = 1.1907x + 1.8388	y = 1.2549x + 0.635
		R^2 = 0.0253	R^2 = 0.0181	R^2 = 0.0549

Abbreviation: C, Soil organic carbon; MBC, Microbial biomass carbon; MBN, Microbial biomass nitrogen; MBP, Microbial biomass phosphorus; MBC:MBN Ratio, Microbial biomass carbon and Microbial biomass nitrogen ratio; MBN:MBP Ratio, Microbial biomass nitrogen and Microbial biomass phosphorus ratio; MBC:MBP Ratio, Microbial biomass carbon and Microbial biomass phosphorus ratio.
